# Israeli medical education: international perspectives, and reflections on challenges and changes

**DOI:** 10.1186/s13584-016-0124-1

**Published:** 2016-12-16

**Authors:** David R. Katz

**Affiliations:** Emeritus Professor of Immunopathology, Division of Infection and Immunity, University College London, Gower St, London, WC1E6BT UK

## Abstract

Medical education is a dynamic and continually evolving process, some of which is style, and some of which is linked to changing perspectives in medical practice. A paper by Reis et al., taken in conjunction with the recent paper from an ad hoc International Review Committee (Schoenbaum et al.), provides a reflective view of where Israeli medical education stood in 2014 and places it in an international perspective. Reis at al also take this further, showing that in Israel change is occurring as a result of this review and comment on a number of important issues where further reflection, discussion, and work is required.

## Background

Medicine and medical education are not immune to changes in style and fashion. In the UK during the 1980s there was a general demise of the essay question in assessment and its replacement by objective, quantifiable, computer-markable multiple choice questions (MCQs). MCQs and structured skills assessment became the order of the day. As the request on examinations to “write short notes on…” disappeared, older physicians bemoaned that the medical student and junior doctor was becoming less literate, less skilled at the well-crafted report letter to colleagues, and less able to communicate complicated issues intelligibly to patients and their families.

Meantime, a quiet shift was taking place. In the UK this manifested in the junior doctor job application process, where “white space” appeared. Applicants were required to comment in brief and succinct sentences about the experience and qualities which justified their selection for a particular post. A terminology for “white space” evolved: “reflective practice”. This has spread way beyond the undergraduate and junior doctor domains, and is now embedded in continuing education, appraisal, and notably is even mentioned as a factor to be considered when doctors are being scrutinised for their fitness to practice.

Another escalating trend has been an increased reporting requirement for medical establishments, including medical schools and hospitals. Many hours are devoted to preparing these documents, which end up being read by a select few. Surely a wider access for such documents, preferably within a peer review context, is warranted — not only to justify the time and resource expended, but also as part of a professional transparency and sharing obligation?

### Israeli medical education

These thoughts spring to mind when considering Prof Reis and colleagues impressive article published in the Journal [1]. When the Israeli medical schools were required to document their practices in undergraduate education for an ad hoc International Review Committee (IRC) appointed by the Israeli Council for Higher Education (CHE) [2], they engaged in the reflective process with aplomb. A precis of part of the reports that were submitted — which might otherwise have languished unseen except by the IRC - is now published in transparent and citable fashion in the Appendices to the paper by Reis et al.; and there are some telling points. Aspiration to common high standards as judged by attainment in examinations primarily comprised of MCQs has to co-exist with some diversity of approach to assessment. All medical schools agree that medical education is more than simple skills training. Israel needs a better national medical education policy strategy.

Perhaps most important is that the article by Reis et al. as a whole represents reflective practice which is both collaborative and constructive, coming from a committed group of doctors engaged within the Israeli medical schools, and based on the comments within the IRC report. Integrated system based curricula, more self-directed learning, and sharing of best practice and of resources where feasible, are all being embraced.

Although the IRC was constituted to focus in detail on medical education and medical schools in Israel, six of its eight members were non-Israelis; and a key question remains open: how many of the IRCs comments and suggestions are international, rather than Israeli, within this rapidly changing field? Where is the “right” balance for Israel between community and hospital based care, and between medical and social care, and how does the doctor, let alone the doctor as medical educator, straddle both? How do you balance the communication and knowledge skills requirements that patients and their families expect from their doctors? How is the role of the medically qualified teacher and educator to be recognised and rewarded, particularly when the drivers towards service - so easily quantifiable in the short term – are so strong? Surely far more “joined up thinking” between health care providers and education/training requirements is essential?

One obvious area of concern raised by the article by Reis et al. is the balance of medical school applicants, student numbers, student placements and workforce requirements. Was it perhaps the duty of the IRC to raise the question which the authors raise in tactful terms, but do not resolve: can Israel afford to continue to provide medical school places and clinical placements for students from abroad, and in parallel to “remediate” training of Israelis forced to study abroad?

Reading these two papers together poses an additional challenge – again, not necessarily an Israeli one, but one where Israel has a distinctive perspective. The Israeli “medical achievement” over a 60 year period has been remarkable; but perhaps even more remarkable is the incredible power of Israeli research-driven biomedical and data/information technology achievements. Of course the primary remit of the Israeli CHE, of the IRC, and of Israeli medical education is to provide the right type of internationally accreditable practitioner. But, on reflection, an important reason why medical education has begun to evolve internationally in the way that it has recently is that physicians trained in the 21^st^ century need to be critical as well as capable, and have to grapple with a highly dynamic research agenda. In the UK Keogh’s mortality review underlined this, saying that the best treatment is delivered by those clinicians who are engaged in research and innovation. Israeli medical education does indeed promote research already, manifesting most prominently in the form of the thesis requirement before completing a medical degree. However, the question is: how best to translate this aspect of medical education into the essential culture of lifelong enquiry and learning?

## Conclusions

Israeli medical education is internationally recognised as being of a high standard and the IRC was able to provide much positive feedback while highlighting areas where there was scope for change. These changes have begun to be embraced by those responsible, and the recent developments will help to align Israeli medicine more closely with international trends in this field. Furthermore, hopefully they will help to reinforce a culture of questioning and research in medicine. This cultural perspective is an important element in attaining high standards of patient care.

## Abbreviations

CHE: Committee for higher education
IRC: International review committee
